# P-Rex1 Expression in Invasive Breast Cancer in relation to Receptor Status and Distant Metastatic Site

**DOI:** 10.1155/2017/4537532

**Published:** 2017-06-15

**Authors:** Jonathan D. Marotti, Kristen E. Muller, Laura J. Tafe, Eugene Demidenko, Todd W. Miller

**Affiliations:** ^1^Department of Pathology & Laboratory Medicine, Dartmouth-Hitchcock Medical Center, Lebanon, NH, USA; ^2^Comprehensive Breast Program, Dartmouth-Hitchcock Medical Center, Lebanon, NH, USA; ^3^Department of Biomedical Data Science, Geisel School of Medicine at Dartmouth, Hanover, NH, USA; ^4^Department of Molecular & Systems Biology, Geisel School of Medicine at Dartmouth, Hanover, NH, USA

## Abstract

**Background:**

Phosphatidylinositol-3,4,5-trisphosphate-dependent Rac exchange factor 1 (P-Rex1) has been implicated in cancer growth, metastasis, and response to phosphatidylinositol 3-kinase (PI3K) inhibitor therapy. The aim of this study was to determine whether P-Rex1 expression differs between primary and metastatic human breast tumors and between breast cancer subtypes.

**Design:**

P-Rex1 expression was measured in 133 specimens by immunohistochemistry: 40 and 42 primary breast tumors from patients who did versus did not develop metastasis, respectively, and 51 breast-derived tumors from metastatic sites (36 of which had matching primary tumors available for analysis).

**Results:**

Primary breast tumors showed significant differences in P-Rex1 expression based on receptor subtype. ER+ and HER2+ primary tumors showed higher P-Rex1 expression than primary triple-negative tumors. HER2+ metastases from all sites showed significantly higher P-Rex1 expression compared to other metastatic receptor subtypes. Solid organ (i.e., brain, lung, and liver) metastases showed higher P-Rex1 expression compared to bone metastases.

**Conclusions:**

P-Rex1 expression is increased in ER+ and HER2+ breast cancers compared to triple-negative tumors. P-Rex1 may be differentially expressed in metastatic tumors based on site and receptor status. The role of P-Rex1 in the development of breast cancer metastases and as a predictive biomarker of therapeutic response warrants further investigation.

## 1. Introduction

It is well-established that breast cancer is a heterogenous disease comprised of several subgroups. By using estrogen receptor *α* (ER), progesterone receptor (PR), and human epidermal growth factor receptor 2 (HER2) status as a surrogate for gene expression profiling, breast cancers are commonly classified into luminal, HER2+, and triple-negative subtypes [1, 2]. These subgroups vary in gene expression signature and have unique responses to therapy and prognosis [1].

The development of distant metastases in breast cancer remains the main cause of mortality: among women with de novo distant metastatic disease, the overall 5-year survival rate is 26% [3]. However, metastatic patterns and associated clinical outcomes among breast cancer subtypes differ and are largely associated with receptor status. For example, luminal A (ER+ and/or PR+ but HER2−) breast cancers tend to develop bone metastasis with better survival compared to the visceral metastases associated with triple-negative breast cancers (TNBCs, which are ER−, PR−, and HER2−) [4]. In light of these inherent differences in metastatic patterns, a better understanding of the biology, particularly within molecular signaling pathways, may lead to novel therapeutic approaches and enhanced prediction of patient outcomes.

We and others recently proposed that the protein phosphatidylinositol-3,4,5-trisphosphate-dependent Rac exchange factor 1 (P-Rex1) is a potential breast cancer biomarker to predict sensitivity to therapeutic inhibitors of phosphatidylinositol 3-kinase (PI3K) [5, 6]. P-Rex1 is a Rho guanine nucleotide exchange factor that actives Rac GTPases [7] and has been shown to be involved in cancer cell growth, migration, invasion, and metastasis [8–10]. In breast cancer, P-Rex1 is an intermediate in receptor tyrosine kinase (ErbB and IGF-1R) and G protein-coupled receptor (GPCR) signal transduction, is activated by PI3K, and feeds back to regulate the PI3K pathway via the Rac/PAK/RAF/MEK/ERK axis [5, 6, 11, 12]. Prior studies showed that P-Rex1 mRNA is overexpressed in luminal and HER2+ breast cancers, while TNBCs lack significant expression of P-Rex1 [5, 11]. However, the relationship between P-Rex1 protein expression, breast cancer receptor status, and metastatic site remains unclear.

## 2. Materials and Methods

### 2.1. Tissue Specimens

This retrospective study was approved by the Dartmouth Committee for the Protection of Human Subjects. The Dartmouth-Hitchcock Medical Center pathology database was retrospectively searched from 2003 to 2015 to identify primary invasive breast cancers and matched metastases. Pathology reports were reviewed to determine ER, PR, and HER2 status. Cancers were categorized as luminal A (ER+ and/or PR+ but HER2−), luminal B (ER+ and/or PR+ and HER2+), HER2+ (ER− and PR− but HER2+), or triple-negative (ER−, PR−, and HER2−). H&E-stained histologic sections from primary and metastatic tumors were reviewed, and one representative formalin-fixed paraffin-embedded (FFPE) tissue block was selected for immunohistochemistry (IHC) analysis.

### 2.2. Immunohistochemistry and Histoscoring

Tumor tissue sections were cut at 4 *μ*m, mounted on slides, and air-dried at room temperature. An automated protocol was performed using the Leica Biosystems BOND RX including bake/dewax and antigen retrieval (BOND Epitope Retrieval 1, cat # AR9961, pH 6.0, 20 min, 100°C). Primary antibody binding utilized the Leica Biosystems BOND Refine Detection kit (DS98000) with a 15-minute incubation and anti-P-Rex1 antibody (Sigma Aldrich, cat # HPA001927, 1 : 100). Visualization was performed using the Leica Refine kit with DAB chromogen and hematoxylin counterstain. Slides were dehydrated, cleared, and mounted with Tissue-Tek Glass Mounting Medium (Sakura). For positive and negative P-Rex1 IHC controls, we used the following: (1) MCF-7 breast cancer cell line- (ATCC-) derived xenografts expressing P-Rex1 and (2) ZR75-1 breast cancer cells (ATCC) transiently transfected with siRNA targeting P-Rex1 (GE Dharmacon) or a nonsilencing control (Qiagen) (Figure S1 in Supplementary Material available online at https://doi.org/10.1155/2017/4537532). The generation of xenografts and transfection of cells was described previously [5]. Two days after transfection, ZR75-1 cells were fixed with formalin, scraped from the dish, mixed with 2% agarose, and formed in 1.5 mL tubes into agarose plugs. Agarose plugs were then embedded in paraffin and processed using the above protocol. Positive and negative control ZR75-1 cells were stained on the same slide. Two pathologists blinded to specimen information independently generated P-Rex1 histoscores. Staining intensity was scored as 0 (negative), 1+ (weak), 2+ (moderate), or 3+ (intense). The entire section was evaluated, and percentage of each staining intensity was estimated. Histoscores ranging from 0 to 300 were then calculated using the following formula: [0 × (% cells scored 0) + 1 × (% cells scored 1+) + 2 × (% cells scored 2+) + 3 × (% cells scored 3+)]. For analysis, the mean of the two pathologists' histoscores was assigned to each specimen.

### 2.3. Statistical Analysis

Correlation of P-Rex1 histoscores between the two pathologists was estimated using Pearson correlation. Unpaired* t*-test and ANOVA with Tukey's honest significant difference (HSD) were used to compare histoscores between groups. Associations between P-Rex1 histoscore and clinical/pathologic features (primary tumor stage, grade, subtype, lymphovascular invasion, lymph node status at initial diagnosis, and whether or not patient eventually developed distant metastasis) were analyzed using the multivariate linear model. *p* ≤ 0.05 was considered significant.

## 3. Results

P-Rex1 expression was evaluated in 133 cases by IHC from 101 patients: 42 primary breast tumors from patients who did not develop distant metastasis within 3–11 years of follow-up (mean = 9.7 years); 40 primary breast tumors from patients who later developed distant metastasis; and 51 breast-derived metastatic tumors, of which 36 had a matching primary breast tumor available for analysis. Tumors from metastatic sites included bone (*n* = 24), brain (*n* = 19), liver (*n* = 4), and lung (*n* = 4). Clinical and pathologic features of the primary breast tumors are detailed in Table 1.

**Figure 1:**
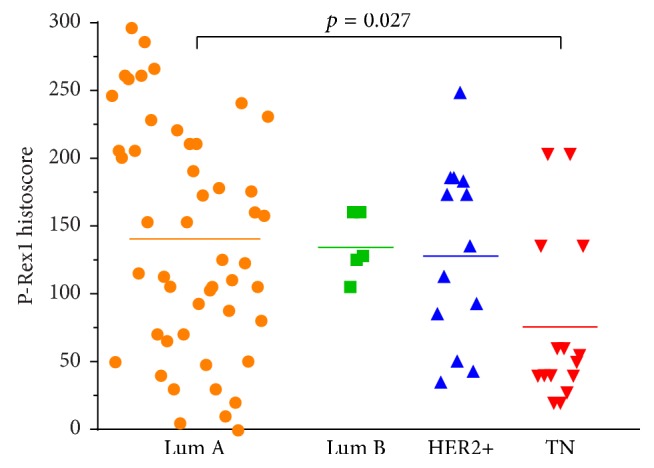
P-Rex1 expression in primary breast tumors. P-Rex1 is highly expressed in luminal (Lum) compared to triple-negative (TN) breast cancers. Each point represents mean P-Rex1 histoscore from two pathologists. Horizontal bars indicate mean histoscore for each receptor subtype. Data were analyzed by ANOVA followed by Tukey's HSD post hoc testing between groups.

**Figure 2:**
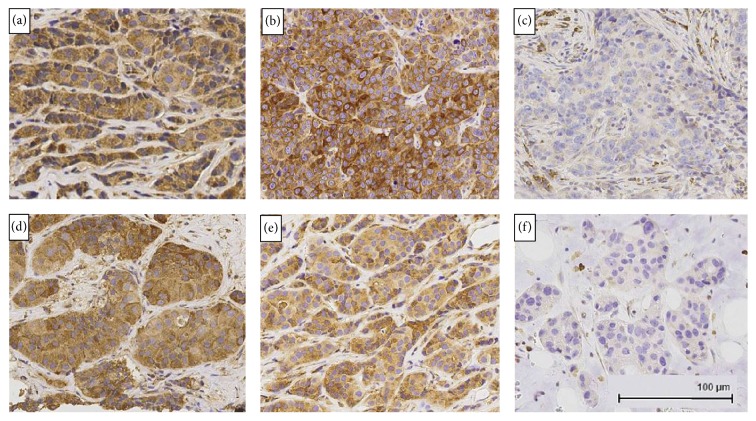
P-Rex1 immunohistochemical expression in primary breast cancers and metastatic sites. (a) Primary ER+ tumor with high expression. (b) Primary HER2+ tumor with high expression. (c) Primary triple-negative tumor with no detectable expression in malignant cells. (d) HER2+ lung metastasis with high expression. (e) ER+/HER2− brain metastasis with high expression. (f) ER+/HER2− bone metastasis with no detectable expression in malignant cells. All images were obtained at 200x magnification.

There was good concordance for P-Rex1 IHC histoscoring between the two pathologists (*R*^2^ = 0.73). Overall, there was a significant difference in P-Rex1 expression in primary breast cancers based on receptor status (ANOVA *p* = 0.019). Primary ER+ (luminal A) (140 ± 84, mean ± SD) and HER2+ (131 ± 66) breast tumors showed higher P-Rex1 expression than primary TNBCs (75 ± 62) (*p* = 0.027 between luminal A and triple-negative tumors, Figures 1 and 2).

**Figure 3:**
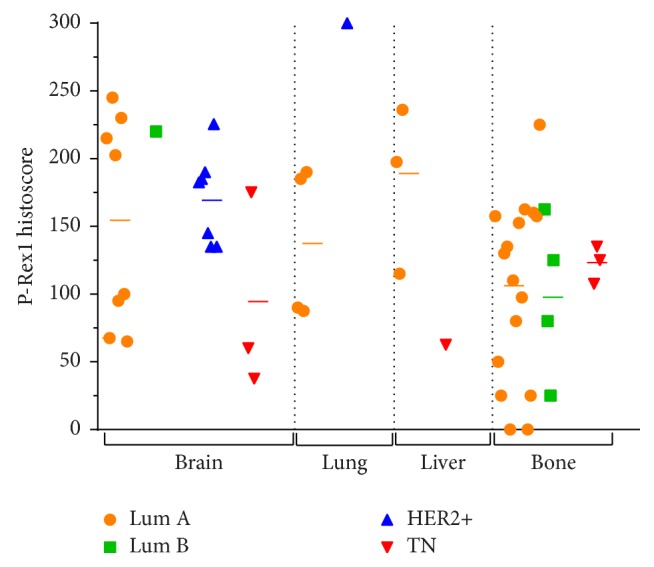
P-Rex1 expression in metastatic breast cancers. P-Rex1 expression is lower in bone metastases compared to brain, lung, and liver metastases. Each point represents mean P-Rex1 histoscore from two pathologists. Horizontal bars indicate mean histoscore for each receptor subtype within each metastatic site.

P-Rex1 expression in tumors from different metastatic sites is shown in Figure 3. P-Rex1 expression in metastases differed based on receptor status (ANOVA *p* = 0.02). HER2+ metastases generally had higher P-Rex1 expression (187 ± 55) compared to luminal A (131 ± 68), luminal B (123 ± 75), and triple-negative tumors (100 ± 49). There was a nonsignificant difference in P-Rex1 expression between metastatic sites when combining all receptor subtypes (ANOVA *p* = 0.07), with a trend toward decreased expression in bone. Analysis of combined brain and visceral (lung, liver) metastases demonstrated higher P-Rex1 expression compared to bone metastases (156 ± 69 versus 106 ± 61, *p* = 0.008), which may be related to the enrichment of HER2+ metastases in the brain and luminal A/B metastases in bone.

**Figure 4:**
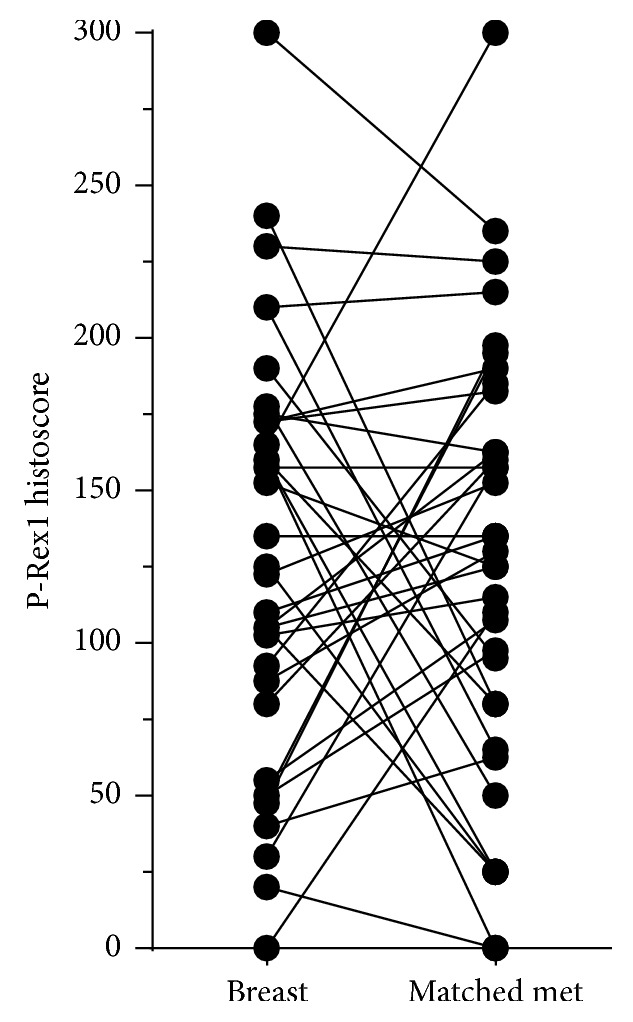
P-Rex1 expression in matched primary breast tumors and metastases. Each point represents mean P-Rex1 histoscore from two pathologists. Lines indicate matching pairs and metastatic tumors.

P-Rex1 expression was similar between primary tumors from patients who did and did not develop distant metastases. Additionally, mean expression of P-Rex1 was not significantly different between metastases and matched (or unmatched) primary breast tumors. For those patients with matched primary cancers and metastases, there was essentially an equal number of cases that demonstrated increased P-Rex1 expression (*n* = 18) and decreased expression (*n* = 15) in the metastases (Figure 4). An increase or decrease in expression was not correlated with metastatic site.

In a multivariate analysis including primary tumor subtype, grade, initial stage, initial lymph node status, lymphovascular invasion in primary tumor, and whether or not a patient eventually developed metastatic disease (Figure S2), the associations between P-Rex1 expression and molecular subtype (*p* = 0.04) and P-Rex1 and tumor grade (*p* = 0.006) remained statistically significant.

## 4. Discussion

In this study, we established that P-Rex1 is differentially expressed in human breast cancers based on receptor status. Primary ER+ (luminal A) and HER2+ breast cancers had the highest P-Rex1 expression, while triple-negative tumors had lower levels. These findings are in alignment with the few prior studies that have examined P-Rex1 expression in human breast cancers, including our prior report that demonstrated a correlation between P-Rex1 and ER in primary breast tumors and breast cancer cell lines [5] Sosa et al. evaluated P-Rex1 mRNA levels in a gene expression microarray data set and reported elevated expression levels in primary luminal breast cancers, but low levels in basal-like (triple-negative) breast cancers [11]. They also found a positive correlation between P-Rex1 and ER mRNA and between P-Rex1 and HER2 mRNA in primary breast tumors. Barrio-Real et al. described a similar differential P-Rex1 expression pattern in breast cancer subtypes and established methylation as a key regulatory mechanism [12]. Increased P-Rex1 expression in ER positive (luminal) breast tumors was associated with hypomethylation of the* PREX1* promotor, while hypermethylation was observed in basal-like breast cancers [12]. Sosa et al. also evaluated P-Rex1 protein expression by IHC in primary tumors that did versus did not ultimately metastasize. P-Rex1 expression was higher in primary tumors that developed metastases relative to those that did not; however, the receptor status of those specimens was not described [11]. In our analysis, we did not detect a difference in P-Rex1 expression between primary breast tumors that did versus did not metastasize.

P-Rex1 is a mediator of tumor progression in models of prostate cancer and melanoma. Utilizing prostate cancer cell lines and a mouse xenograft model, Qin et al. demonstrated that P-Rex1 acts as detector of chemotactic signals with the subsequent activation of Rac to promote metastasis [9]. In melanoma models, Lindsay et al. determined that P-Rex1 deficiency suppresses melanoblast migration and development of metastases [10]. The exact role of P-Rex1 in the development of breast cancer metastasis remains unclear, although high expression levels have been shown to be associated with poor patient outcome [13]. Recent evidence has linked P-Rex1 with the expression of matrix metalloprotease 10, which is involved with remodeling of the extracellular matrix and may enhance breast cancer cell invasiveness [14].

Our study uniquely examined multiple distant metastatic sites, many of which also had a matched primary breast tumor available for analysis. HER2+ breast cancer metastases, which were from either brain or lung, had the highest levels of P-Rex1 expression. These observations lend further support to the role of P-Rex1 in HER2-driven oncogenic signaling [11, 15].

Bone metastases had the lowest levels of P-Rex1 expression, despite the majority of bone metastases being of the luminal (ER+) subtypes. Given that high P-Rex1 levels have been associated with poorer outcomes, the lower P-Rex1 expression in bone metastases potentially correlates with the prognosis related to metastatic site; patients with visceral metastases tend to have worse survival. It should be noted that many of the bone metastases were sampled via core biopsy and underwent decalcification, typically with ethylenediaminetetraacetic acid (EDTA). The impact of decalcification on P-Rex1 IHC expression is largely unknown and might have affected interpretation of expression levels in bone specimens.

The definition of the luminal B subtype continues to evolve, with recent evidence suggesting the existence of both luminal B/HER2+ and luminal B/HER2− subtypes [2]. The luminal B/HER2+ subtype expresses ER and/or PR plus HER2, while the luminal B/HER2− subtype is commonly defined as ER+ and HER2−, and either PR ≤ 20% or Ki-67 ≥ 14%. We chose to identify those tumors expressing ER and/or PR and HER2 as luminal B tumors. In our cohort of primary breast cancers that did not develop metastases, there were no luminal B/HER2+ tumors, and only 2/23 luminal A tumors were high-grade (potentially luminal B/HER2− cases). The relationship between P-Rex1 expression and the proposed different luminal B subtypes should be examined in studies with larger case numbers.

## 5. Conclusions

This study independently corroborates the P-Rex1 expression patterns that have been reported in breast cancer cell lines and at the mRNA level in human tumors. Using a commercially available antibody on a cohort of routine clinical cases, we confirmed that P-Rex1 is increased in ER+ and HER2+ breast cancers compared to triple-negative tumors. Furthermore, our data suggest that P-Rex1 may be differentially expressed in metastatic tumors based on site and receptor status. Provided that increased P-Rex1 expression might predict sensitivity to PI3K inhibitors in breast cancer [5, 6], the role of P-Rex1 in the development of breast cancer metastasis and therapeutic sensitivity warrants further investigation.

## Supplementary Material

Figure S1: P-REX1 immunohistochemical controls. A) Positive control; MCF-7 breast cancer xenograft that expresses P-Rex1 (x155). B) Positive control; ZR75-1 breast cancer cells transfected with non-silencing control siRNA (x145). C) Negative control; ZR75-1 breast cancer cells transfected with siRNA targeting P-Rex1 (x145). Figure S2: Univariate and multivariate analyses of associations between clinical and pathologic features and P-Rex1 histoscore. Based on univariate analyses (shown below), we found that primary tumor subtype and grade were significantly associated with P-Rex1 histoscore; these covariates were included in a multivariate linear model as follows: Subtype: TN vs. other, Grade: Intermediate vs. other. Primary tumor subtype (*p* = 0.04) and grade (*p* = 0.006) remained significantly associated with P-Rex1 histoscore in the multivariate analysis (multiple *R*^2^ = 0.16, *p* = 0.001).

## Figures and Tables

**Table 1:** Clinical and pathologic features of primary breast tumors.

	Primary tumors from patients who did not develop distant metastasis			Primary tumors from patients who developed distant metastasis			
	Luminal A (*n* = 23)	HER2 (*n* = 9)	TN (*n* = 10)	Luminal A (*n* = 26)	Luminal B (*n* = 5)	HER2 (*n* = 4)	TN (*n* = 5)
*Histology*							
IDC	22 (96%)	9 (100%)	10 (100%)	20 (77%)	3 (60%)	4 (100%)	5 (100%)
ILC	0	0	0	5 (19%)	1 (20%)	0	0
Other	1 (4%)	0	0	1 (4%)	1 (20%)	0	0
*Grade*							
Low	14 (61%)	0	0	2 (8%)	0	0	0
Intermediate	7 (30%)	1 (11%)	0	18 (70%)	3 (60%)	0	0
High	2 (9%)	8 (89%)	10 (100%)	6 (23%)	2 (40%)	4 (100%)	5 (100%)
*LVI*							
Present	2 (9%)	1 (11%)	2 (20%)	13 (50%)	4 (80%)	3 (75%)	3 (60%)
Absent	21 (91%)	8 (89%)	8 (80%)	13 (50%)	1 (20%)	1 (25%)	2 (40%)
*Lymph node involv.*							
Present	5 (22%)	0	0	13^*∗*^ (68%)	4^*∗*^ (100%)	3^*∗*^ (100%)	2 (40%)
Absent	18 (78%)	9 (100%)	10 (100%)	6^*∗*^ (32%)	0	0	3 (60%)
*Tumor size*							
≤2 cm	22 (96%)	5 (56%)	4 (40%)	6^*∗*^ (24%)	2 (40%)	0	2 (40%)
2.1–5 cm	1 (4%)	3 (33%)	6 (60%)	14^*∗*^ (58%)	1 (20%)	2 (50%)	2 (40%)
>5 cm	0	1 (11%)	0	5^*∗*^ (20%)	2 (40%)	2 (50%)	1 (20%)

^*∗*^Data not available for all cases within subgroup. TN, triple-negative; IDC, invasive ductal carcinoma; ILC, invasive lobular carcinoma; LVI, lymphovascular invasion; involv., involvement.
