# Supplementary material for: P-Rex1 Expression in Invasive Breast Cancer in relation to Receptor Status and Distant Metastatic Site
Source: Int J Breast Cancer. 2017 Jun 15;2017:4537532. doi: 10.1155/2017/4537532 (PMC5494073; doi:10.1155/2017/4537532)

**Figure S1.** P-REX1 immunohistochemical controls. A**)** Positive control**;** MCF-7 breast cancer xenograft that expresses P-Rex1 (x155). B) Positive control; ZR75-1 breast cancer cells transfected with non-silencing control siRNA (x145). C) Negative control; ZR75-1 breast cancer cells transfected with siRNA targeting P-Rex1 (x145).

**
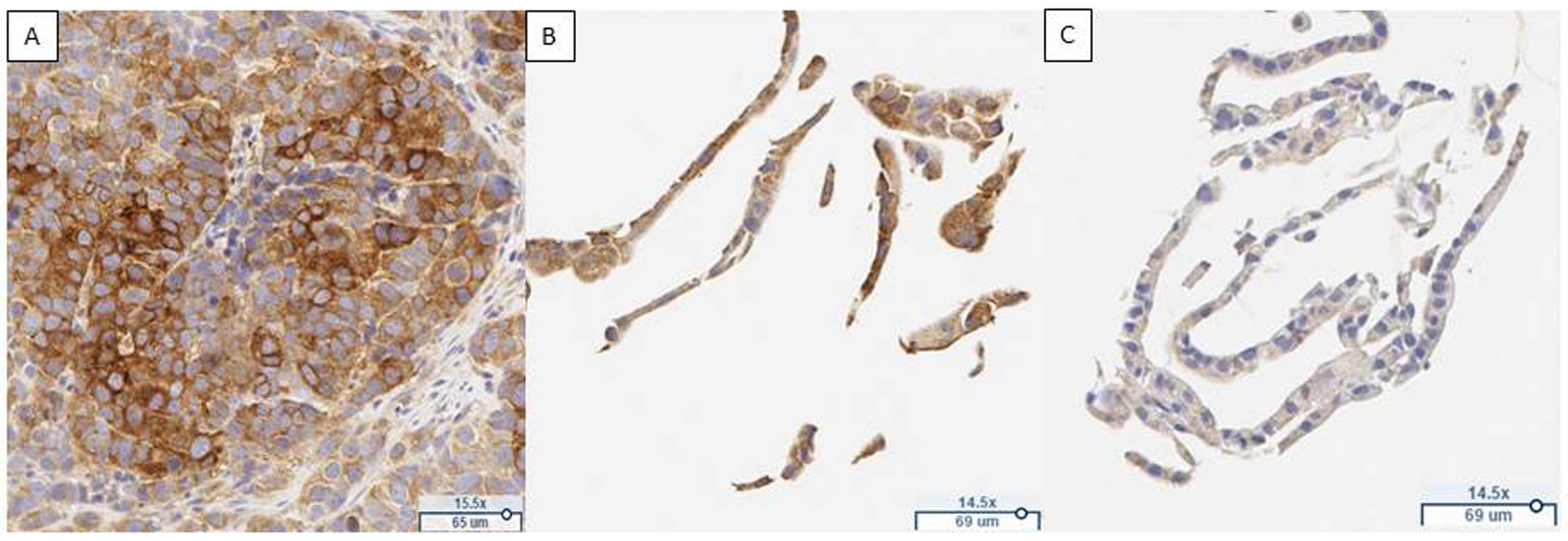
**

**Figure S2.** Univariate and multivariate analyses of associations between clinical and pathologic features and P-Rex1 histoscore. Based on univariate analyses (shown below), we found that primary tumor subtype and grade were significantly associated with P-Rex1 histoscore; these covariates were included in a multivariate linear model as follows:

Subtype: TN vs. other

Grade: Intermediate vs. other

Primary tumor subtype (*p*=0.04) and grade (*p*=0.006) remained significantly associated with P-Rex1 histoscore in the multivariate analysis (multiple *R*^2^=0.16, *p*=0.001)


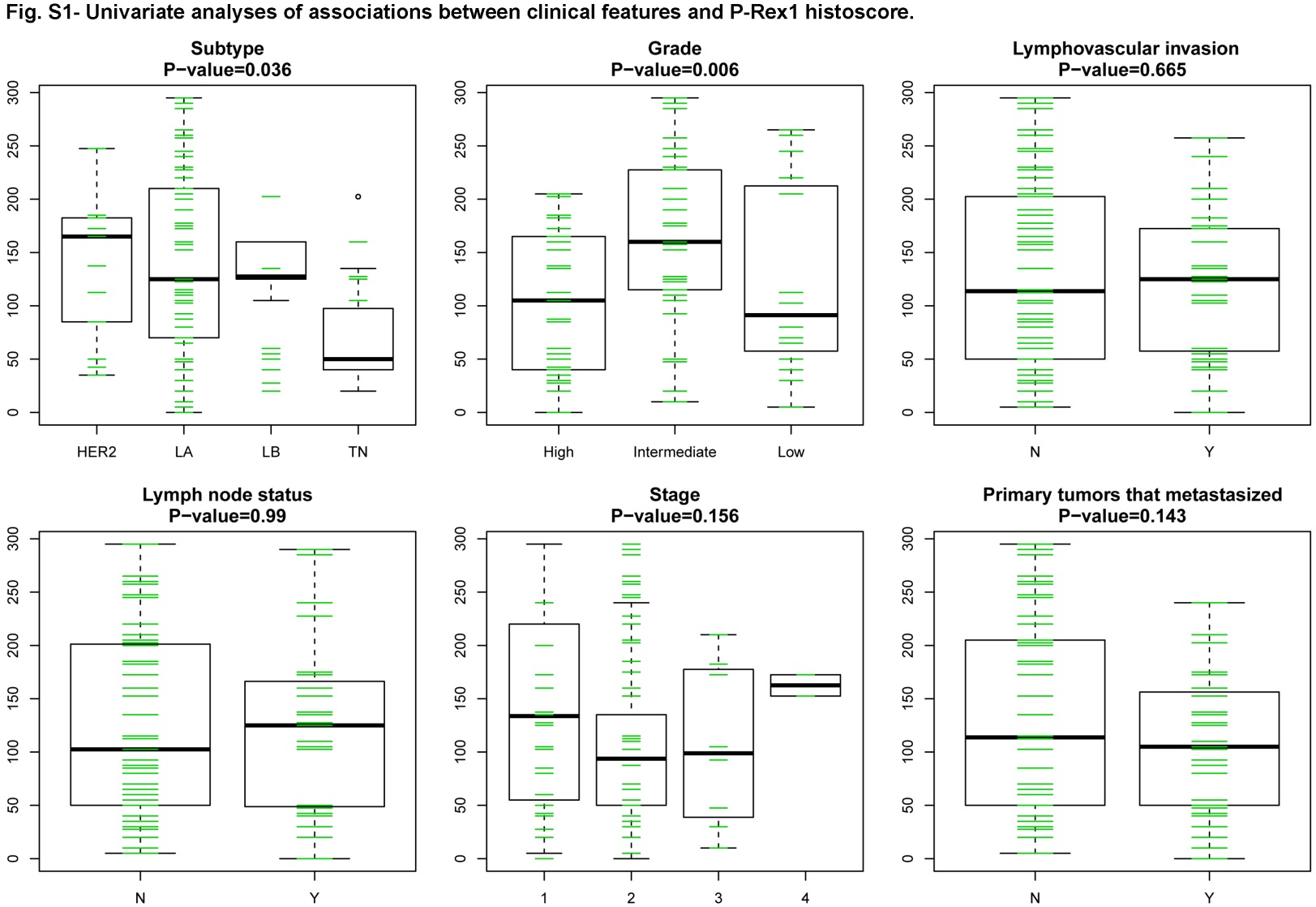

Supplement: Supplementary file 1 — Figure S1: P-REX1 immunohistochemical controls. A) Positive control; MCF-7 breast cancer xenograft that expresses P-Rex1 (x155). B) Positive control; ZR75-1 breast cancer cells transfected with non-silencing control siRNA (x145). C) Negative control; ZR75-1 breast cancer cells transfected with siRNA targeting P-Rex1 (x145). Figure S2: Univariate and multivariate analyses of associations between clinical and pathologic features and P-Rex1 histoscore. Based on univariate analyses (shown below), we found that primary tumor subtype and grade were significantly associated with P-Rex1 histoscore; these covariates were included in a multivariate linear model as follows: Subtype: TN vs. other, Grade: Intermediate vs. other. Primary tumor subtype (p = 0.04) and grade (p = 0.006) remained significantly associated with P-Rex1 histoscore in the multivariate analysis (multiple R2 = 0.16, p = 0.001). [file 4537532.f1.docx]
